# Cryoelectrospun Elastin-Alginate Scaffolds Support In Vitro 3D Epithelial-Stromal Cocultures for Salivary Tissue Engineering

**DOI:** 10.3390/gels11120998

**Published:** 2025-12-11

**Authors:** Pujhitha Ramesh, James Castracane, Melinda Larsen, Deirdre A. Nelson, Susan T. Sharfstein, Yubing Xie

**Affiliations:** 1 Department of Nanoscale Science and Engineering, College of Nanotechnology, Science, and Engineering, University at Albany, State University of New York (SUNY), Albany, NY 12203, USA; jim.castracane@gmail.com (J.C.); ssharfstein@albany.edu (S.T.S.); 2 Department of Biological Sciences, University at Albany, State University of New York (SUNY), Albany, NY 12222, USA; mlarsen@albany.edu (M.L.); dnelson@albany.edu (D.A.N.); 3 The RNA Institute, University at Albany, State University of New York (SUNY), Albany, NY 12222, USA

**Keywords:** cryoelectrospinning, alginate, elastin, extracellular matrix, 3D porous scaffold, 3D coculture, epithelial-stromal coculture, salivary gland tissue engineering

## Abstract

Bioengineered functional salivary tissues can advance regenerative therapies, preclinical drug testing, and the fundamental understanding of salivary gland dysfunction. Current salivary tissue models are typically Matrigel-based, hydrogel-based or scaffold-free organoid systems, with limited physiological relevance or mimicry of cell-cell and cell-extracellular matrix (ECM) interactions. We previously developed elastin-alginate cryoelectrospun scaffolds (CES) that resemble the topography and viscoelastic properties of healthy salivary ECM, and validated their potential for stromal cell culture, delivery, and in vitro fibrosis modeling. Here, we evaluated the utility of CES to support 3D cocultures of salivary gland epithelial and mesenchymal cells in vitro. We compared CES with honeycomb-like topography (CES-H) to densely packed electrospun nanofibers (NFs) and CES with fibrous topography (CES-F) for their ability to support SIMS epithelial cell attachment, morphology, 3D clustering, phenotype and organization into distinct clusters when cocultured with stromal cells. Both CES-F and CES-H supported epithelial cell attachment and clustering; in particular, CES-H most effectively supported the self-organization of epithelial and stromal cells into distinct 3D clusters resembling the structure of native salivary tissue. Stromal cells were essential for maintaining the phenotype of epithelial cells cultured on CES-H, laying the foundation for the development of in vitro tissue models.

## 1. Introduction

The salivary gland is essential for oral and digestive health but can be impacted by pathologies, leading to salivary gland hypofunction, hyperfunction, and cancer [1]. Current treatment options for salivary gland dysfunction are largely palliative [2] and offer minimal relief, highlighting the need for curative therapies based on a comprehensive understanding of the fundamental mechanisms governing disease pathology.

Animal models, Matrigel^TM^- or other hydrogel-based in vitro cell culture systems, and organoids are currently employed to study both salivary gland development and disease mechanisms [3,4,5,6,7,8,9,10,11,12]. While mouse models are often employed to study disease pathways and test therapeutic candidates, they are expensive, time-consuming, and poorly reflect human physiology, resulting in high failure rates for therapeutic candidates in clinical trials [13,14]. Matrigel-based models are relatively easily established, as Matrigel contains a growth factor cocktail that can support most primary cell types, including salivary epithelial and stromal cells [3,4,5,6]. However, its batch-to-batch variability, sarcoma tumor-derived and ill-defined composition can skew study results and make it unsuitable for clinical translation [15,16]. Several hydrogel-based in vitro salivary gland models have been developed to attempt to replace Matrigel-based cultures [7,8]; however, in these systems, the bulk hydrogel completely encapsulates salivary epithelial and stromal cells, failing to mimic the porous native extracellular matrix (ECM) architecture, and inadequately mimicking salivary ECM mechanical properties and cell-ECM interactions. Scaffold-free organoid models avoid the complexity of incorporating physiologically relevant biomaterial scaffolds but tend to mimic the early gland developmental stages rather than the complexity of adult gland cell-cell interactions and cell-ECM interactions [17]. While organoids are good candidates to study developmental disorders and cancers, they may not mimic other adult human pathologies [17,18]. Thus, there is a need to develop physiologically relevant in vitro models that comprehensively mimic the native 3D cell organization, cell-cell communication, cell-ECM interactions, and cell phenotypes for translational preclinical research. Such functional 3D tissue models could also serve as platforms for regenerative, implantable therapies.

We previously developed 3D elastin-alginate cryoelectrospun scaffolds (CES) with fibrous (CES-F) and honeycomb-like (CES-H) topographies and showed that CES-H mimics the porosity, pore size, and viscoelasticity of native salivary ECM [19,20]. Elastin and alginate were selected as the biomaterials to mimic the elastic and viscous nature of stromal matrices to support stromal cell culture. CES-H demonstrated ~120 Pa indentation modulus and 150–200 s relaxation half-time, comparable to SDS-decellularized mouse salivary glands [19]. Functionally, CES-H supported the maintenance of mesenchymal stromal cells (MSCs), preserving their innate phenotype [20,21]. MSCs on CES-H exerted therapeutic, anti-fibrotic effects on fibrotic myofibroblasts in a preclinical in vitro model [21], ameliorating fibrosis, the underlying pathology in salivary gland hypofunction. Additionally, we demonstrated that MSC-CES-H implants were biocompatible, eliciting neither inflammatory nor fibrotic effects in mice [21].

To address some of the limitations of current in vitro salivary tissue models by applying CES for salivary tissue engineering, we explored whether CES can support salivary epithelial monoculture and epithelial-stromal coculture. Since stromal cells are key regulators of salivary epithelial cell health in vivo and in vitro [22,23], we maintained the elastin-alginate biomaterial composition to provide a compliant structural framework that supports stromal culture, which may, in turn, enhance epithelial cultures. In vivo, epithelial cells reside on densely packed nanofibrous basement membranes with kPa–MPa stiffness [24,25], which regulate their function and phenotype, whereas stromal cells reside in loosely packed, viscoelastic, reticulated, porous matrices with sub-kPa to low kPa stiffness [26,27,28]. In vitro, epithelial cells are difficult to grow on tissue culture plastic when compared to stromal cells, due to limited expansion potential, senescence, and transdifferentiation, necessitating growth in Matrigel and/or with special media formulations containing specific growth factors or small molecules [29,30,31]. We hypothesized that 3D matrices integrating both basement membrane and stromal ECM features would facilitate the growth of 3D epithelial-stromal cocultures. To test this hypothesis, in this study, we evaluate the role of elastin-alginate scaffold topography on epithelial cell phenotype and cell organization in 3D salivary epithelial cultures by comparing honeycomb-like CES-H with densely packed electrospun nanofibers (NFs) and fibrous CES-F. We then evaluate the role of stromal fibroblast cells in combination with scaffold topography (CES-F vs. CES-H) in regulating epithelial phenotype and 3D epithelial and stromal cell organization. We used SIMS cells, an established mouse submandibular salivary gland ductal epithelial line [32], as the salivary epithelial model, for their ability to form polarized epithelial structures [31], and NIH 3T3 fibroblasts, a widely used fibroblast line [33], to establish stromal cultures. Overall, we assess the potential of CES to bioengineer 3D salivary epithelial-stromal cocultures towards functional salivary tissue engineering for preclinical research and regenerative medicine strategies.

## 2. Results

### 2.1. Characterization of Scaffold Topography Prior to Cell Seeding

**Figure 1:**
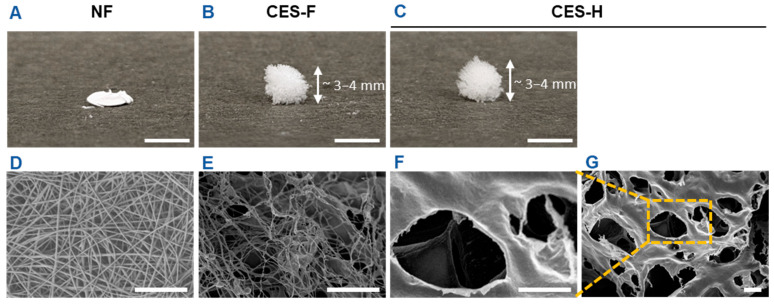
Benchtop photos and SEM images depicting the scaffold dimensions and topography. (**A**,**D**) Electrospun nanofibers (NF). (**B**,**E**) Cryoelectrospun scaffolds with fibrous topography (CES-F). (**C**,**F**,**G**) Cryoelectrospun scaffolds with honeycomb topography (CES-H). (**A**–**C**) Benchtop photos. Scale bar = 5 mm. (**D**–**G**) SEM images. Scale bar = 10 μm.

First, we fabricated electrospun nanofiber (NF) mats and cryoelectrospun scaffolds with fibrous (CES-F) or honeycomb (CES-H) topography from elastin-alginate biopolymers to mimic the compliant nature of stromal ECM and/or fibrous topography of epithelial basement membranes as previously described [19,20,34]. NF mats were fabricated to resemble the densely packed, fibrous nature of basement membranes. CES-F were designed to mirror the fibrous topography of basement membranes and loosely packed nature of stromal ECM, whereas CES-H were designed to resemble the porous and loosely packed nature of stromal ECM. Scanning electron microscopy (SEM) revealed that NF mats were composed of densely packed, ~200 nm ± 5 nm diameter nanofibers with <2 µm pores (Table 1), forming a 2.5D mat (Figure 1A,D). CES-F showed loosely packed ~178 nm ± 80 nm diameter nanofibers with <5 µm pores (Table 1), forming a taller 3D structure (Figure 1B,E). In contrast, CES-H displayed a honeycomb-like, reticulated topography (Figure 1C,F,G), with larger backbones (~4.72 ± 3.86 µm width) and pores (~15–25 µm diameter) (Table 1), forming a 3D scaffold similar in height to CES-F (Figure 1B,C).

### 2.2. Cryoelectrospun Scaffolds with Honeycomb and Fibrous Topography Promote Clustered Salivary Epithelial Cell Growth

**Figure 2:**
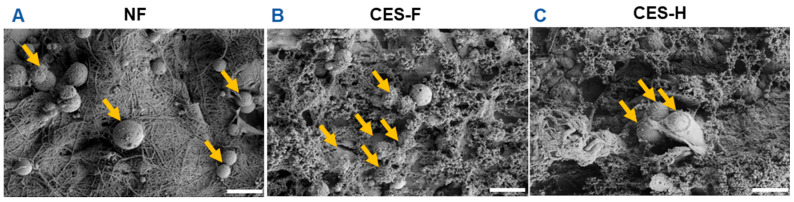
Effects of scaffold topography on SIMS epithelial cell morphology and clustering. SEM images showed that SIMS epithelial cells remained isolated and rounded on conventionally electrospun nanofibers (NF) (**A**) and formed cell clusters on cryoelectrospun scaffolds with fibrous topography (CES-F) (**B**) and honeycomb topography (CES-H) (**C**) after 1 day in culture. Arrows indicate cells. Scale bar = 25 μm.

To assess how scaffold topography affects epithelial attachment and clustering, SIMS salivary epithelial cells were cultured on NF, CES-F, and CES-H scaffolds. After 1 day of culture, SIMS cells on scaffolds were imaged using SEM. On NF mats, epithelial cells remained rounded (Figure 2A), whereas on CES-F and CES-H scaffolds, they exhibited distinct cell bodies and formed cell clusters (Figure 2B,C).

### 2.3. Cryoelectrospun Scaffolds with Honeycomb Topography Enable Deep Penetration of 3D Salivary Epithelial Cell Clusters and Distinct 3D Epithelial-Stromal Organization in Cocultures

**Figure 3:**
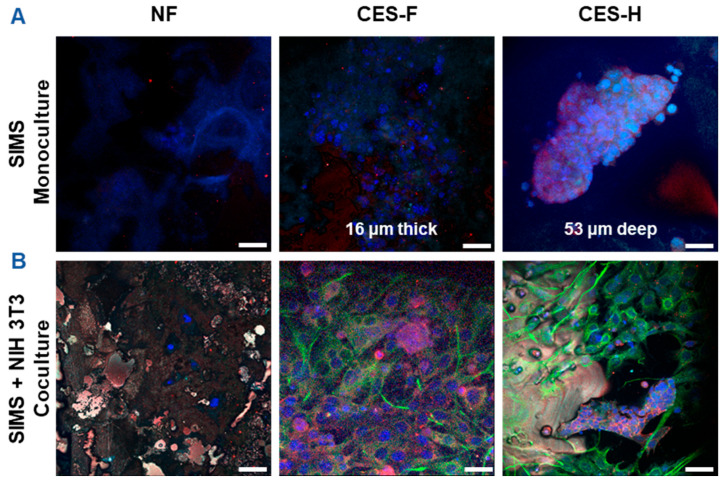
Confocal images showing effects of scaffold topography on 3D organization of SIMS epithelial cells in monoculture and coculture with NIH 3T3 fibroblasts. (**A**) SIMS monoculture on scaffolds. (**B**) SIMS epithelial cells cocultured with NIH 3T3 fibroblasts on scaffolds. Cells grown on NF mats (left panel), cryoelectrospun scaffold with fibrous topography (CES-F) (middle panel), and cryoelectrospun scaffold with honeycomb topography (CES-H) (right panel) were immunostained with epithelial junction markers (E-cadherin in red and ZO-1 in cyan) and mesenchymal marker (vimentin in green), along with DAPI-stained nuclei (in bluish purple) on day 4. Scale bar = 25 μm.

To assess how scaffold architecture affects 3D growth and penetration of epithelial cell clusters and maintenance of epithelial cell morphology, SIMS salivary epithelial cells (monoculture) were cultured on NF, CES-F, and CES-H scaffolds for 4 days (Figure 3A). To examine scaffold-topography-dependent stromal-epithelial organization, SIMS cells were cocultured with NIH 3T3 fibroblasts, a stromal model, on each scaffold type. We anticipated that appropriate topography would result in distinct 3D cell clusters, similar to stromal and parenchymal compartmentalization observed in vivo [35]. NIH 3T3 fibroblasts were seeded first and allowed to expand for 2 days to act as stromal support for SIMS cells, which were subsequently seeded and cocultured with the fibroblasts for 4 additional days (Figure 3B).

SIMS cells were identified by immunostaining for intercellular epithelial junction markers, E-cadherin and Zona occludin-1 (ZO-1) [36], while NIH 3T3 cells were identified by immunostaining for vimentin, a mesenchymal phenotype marker [37]. 4′,6-diamidino-2-phenylindole (DAPI) was used for nuclear staining for both cell types. NF mats showed poor cell attachment in both SIMS monocultures and SIMS-NIH 3T3 cocultures (Figure 3A,B, left panels), with observed blue fluorescence primarily representing autofluorescence. On CES-F, SIMS monocultures remained on the scaffold surface and formed thin epithelial sheets (Figure 3A, middle panel), whereas on CES-H, they penetrated the scaffold, forming deep 3D clusters (Figure 3A, right panel). In cocultures, CES-F showed random cell attachment (Figure 3B, middle panel), whereas CES-H supported the self-organization of epithelial and stromal cells into distinct clusters (Figure 3B, right panel).

### 2.4. Stromal Cells on Cryoelectrospun Scaffolds with Honeycomb Topography Facilitate Phenotypic Maintenance of Salivary Epithelial Cells

**Figure 4:**
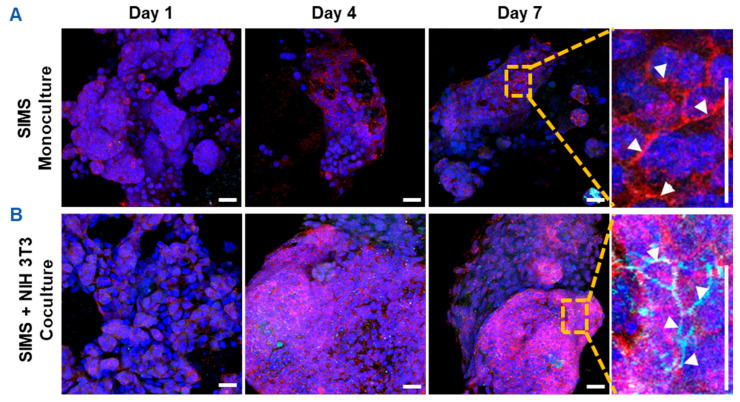
Confocal images showing effects of stromal support on epithelial cell phenotype on cryoelectrospun scaffolds with honeycomb topography. (**A**) Monoculture of SIMS epithelial cells on CES-H demonstrated 3D cell growth with membrane-localized expression of E-cadherin but not ZO-1. (**B**) Coculture of SIMS epithelial cells with NIH 3T3 fibroblasts promotes membrane-localized expression of both epithelial markers, E-cadherin and ZO-1. Right panel: enlarged view (7× magnification) of cells cultured on CES-H on day 7 (vertical scale bar). Red, E-cadherin. Cyan, ZO-1. Green, vimentin. Bluish purple, DAPI-stained nuclei. Arrowheads point to membrane-localized expression of E-cadherin and/or ZO-1. Scale bar = 25 μm.

Since CES-H promoted stromal-epithelial organization into distinct 3D clusters (Figure 3B, right panel), we further examined whether stromal cells influence epithelial phenotype over 1, 4, and 7 days of culture. SIMS epithelial cells were cultured alone or cocultured with NIH 3T3 fibroblasts on CES-H scaffolds. Using confocal microscopy, epithelial phenotype was assessed by E-cadherin and ZO-1 expression and localization, as E-cadherin assembled at cell membranes is indicative of formation of adherens junctions and ZO-1 of tight junctions between epithelial cells [36]. Stromal cells were identified by vimentin immunostaining; all nuclei were counterstained with DAPI. In monoculture, SIMS cells on CES-H showed expression of E-cadherin by day 4 but little ZO-1 (Figure 4A). In coculture with NIH 3T3 cells on CES-H, SIMS cells formed robust 3D clusters with strong membrane localization of both epithelial markers from day 4 to 7 (Figure 4B). These findings demonstrate that stromal cells cultured on CES-H play a critical role in enhancing epithelial cell phenotype and polarization compared to epithelial monocultures.

## 3. Discussion

Matrix topography, architecture, composition and mechanical properties govern cell-matrix interactions and, together with cell-cell communication, regulate cell function in health and disease [26,38,39,40]. Replicating these cues is essential for developing physiologically relevant tissues for preclinical research or regenerative therapy. In vitro cell culture systems, aiming to recapitulate the epithelial lining or epithelial-stromal interface, typically grow epithelial cells on dense basement membrane-like matrices or encapsulate epithelial and stromal cells in hydrogels [41,42,43]. While stiff nanofiber mats composed of water-insoluble biomaterials support robust epithelial cell growth [41,44], we hypothesized that matrices incorporating both epithelial and stromal ECM properties would better support epithelial-stromal cocultures. We therefore compared three scaffold types representing distinct epithelial and/or stromal ECM properties.

Densely packed electrospun nanofibers (NFs) fabricated from an elastin-alginate solution resembled the structural architecture of epithelial basement membranes and the compliant nature of stromal ECM but poorly supported epithelial cell attachment in both monocultures and epithelial-stromal cocultures (Figure 2A and Figure 3A,B, left panels). This result was consistent with our prior work with NIH 3T3 stromal cells, where the alginate in these NF scaffolds swelled to form a unified hydrogel layer [19], limiting cell adhesion and viability. Therefore, densely packed nanofibers may be suitable for epithelial culture, only when composed of insoluble, stiff biomaterials that preserve the nanofibrous topography upon hydration.

Cryoelectrospun scaffolds with fibrous topography (CES-F) combining the fibrous nature of basement membranes, and the loosely packed, compliant 3D structure of stromal ECM supported epithelial cell attachment and morphology (Figure 2B and Figure 3A,B, middle panels). Unlike NF mats, CES-F retained topography upon hydration, permitting cell adhesion and clustering. However, its fibrous mesh limited vertical cell penetration, leading to horizontal 1- to 3-layer thick cell sheets in both mono- and cocultures (Figure 3A,B, middle panels), and a random mix of epithelial and stromal cells in cocultures rather than distinct 3D clusters. Thus, CES-F supports epithelial attachment but not 3D epithelial-stromal organization.

Cryoelectrospun scaffolds with a honeycomb-like, reticulated topography (CES-H) supported epithelial attachment, clustering, and at least 50 µm deep penetration (Figure 2C and Figure 3A right panel). However, CES-H alone only maintained E-cadherin expression and localization, and not ZO-1 expression (Figure 4A), possibly because CES-H does not mimic the structure or complex composition of epithelial basement membranes. Coculture with stromal cells on CES-H restored membrane-localized E-cadherin and ZO-1 expression (Figure 4B) in SIMS cells, highlighting the necessity of stromal support for maintaining epithelial phenotypes on CES-H. We previously demonstrated that CES-H recapitulates the reticulated topography, sub-kPa stiffness and slow relaxation dynamics (150–200 s relaxation half-time) of native salivary ECM [19] and supports stromal cells while suppressing fibrotic differentiation [21]. Furthermore, we showed that CES-H suppressed the fibrotic phenotype in myofibroblasts in vitro [21]. The physical and mechanical properties of CES-H might be best suited for optimal stromal culture, which likely enabled NIH 3T3 fibroblasts to sustain the epithelial phenotype of SIMS cells through paracrine signaling or ECM deposition. Stromal cells regulate salivary epithelial function via growth factors (e.g., fibroblast growth factors, bone morphogenetic proteins and macrophage colony stimulating factor), ECM modulation and direct epithelial-stromal interactions [22,23,45,46,47]. However, the specific mechanisms by which stromal cells on CES-H promote the epithelial phenotype remain to be investigated. CES-H also promoted the most physiologically relevant 3D organization of stromal and epithelial clusters (Figure 3A,B middle vs. right panels). The larger (~15–25 µm) pores likely enabled deeper penetration and spatial segregation of clusters, mimicking native stromal–parenchymal architecture.

These foundational studies demonstrate that matrices that support stromal cell growth and phenotype maintenance might best facilitate healthy stromal-epithelial cocultures. While densely packed nanofiber mats or loosely packed fibrous meshes fabricated with stiff insoluble biomaterials (e.g., collagen, laminin) may enable cell attachment and growth of epithelial and stromal cells, they risk inducing stromal fibrosis and, thereby, epithelial-to-mesenchymal transition [48,49,50,51]. Such stiff scaffolds may be better suited for modeling diseased or fibrotic tissue, but not healthy tissue.

Further work is necessary to validate the utility of CES-H to support more physiologically relevant parenchymal-stromal cocultures, using primary or stem cell-derived epithelial and stromal cells, to evaluate acinar differentiation and secretory function, as softer matrices promote salivary morphogenesis [52]. Given the soft ECM-like properties of CES-H, they may also be adaptable for stromal–parenchymal cocultures of other soft tissues such as lung, liver, and pancreas, building on these foundational studies.

## 4. Conclusions

Elastin-alginate cryoelectrospun scaffolds with honeycomb-like topography (CES-H) supported 3D growth and organization of SIMS epithelial cells in both monocultures and cocultures with NIH 3T3 fibroblasts. Cells penetrated these scaffolds and self-organized into tissue-like structures with characteristic epithelial-like organization. In particular, these scaffolds facilitated native tissue-like, distinct 3D stromal and epithelial cluster formation and stromal-epithelial interaction, which enhanced epithelial phenotype. These findings provide a foundation for future applications of honeycomb-like cryoelectrospun scaffolds for in vitro organ modeling and in vivo soft tissue regeneration.

## 5. Materials and Methods

### 5.1. Materials

Scaffolds were fabricated using soluble bovine neck elastin (ES12; Elastin Products Co., Owensville, MI, USA), alginate, and 400 kD polyethylene oxide (PEG-400kD) from Sigma-Aldrich (St. Louis, MO, USA). Scaffold-crosslinking reagents were N-hydroxysuccinimide (NHS; Thermo Fisher Scientific, Waltham, MA, USA), ethyl dimethylaminopropyl carbodiimide (EDC), and calcium chloride dihydrate (both from Sigma-Aldrich). Cell culture reagents were high-glucose Dulbecco’s Modified Eagle’s Medium (DMEM), heat-inactivated fetal bovine serum (FBS), and penicillin-streptomycin (10,000 U/mL penicillin and 10,000 µg/mL streptomycin) all from Thermo Fisher Scientific, or Antibiotic-Antimycotic Solution (10,000 U/mL penicillin, 10,000 µg/mL streptomycin and 25 µg/mL amphotericin B; R&D Systems, Minneapolis, MN, USA). Multi-well, cell culture plates were coated with the ultra-low adhesion polymer Lipidure (Amsbio; Cambridge, MA, USA). Cell lines for cell culture were NIH 3T3 mouse embryonic fibroblasts sourced from ATCC (CRL-1658, Manassas, VA, USA), and SIMS submandibular salivary ductal epithelial cell line gifted by the late Dr. D. Malamud and Dr. B.M. Laoide. For scanning electron microscopy (SEM) sample preparation, glutaraldehyde, sucrose, phosphate buffer, and hexamethyldisilazane (HMDS) were obtained from Sigma-Aldrich, and ethanol was obtained from Decon Labs (King of Prussia, PA, USA). Primary antibodies used for immunocytochemistry analysis included mouse anti-vimentin (µ-chain specific; clone LN-6; Cat. No. V2258; Sigma-Aldrich), rabbit anti-ZO-1 (Cat. No. 402200; Thermo Fisher Scientific), and mouse anti-E-cadherin (Cat. No. 610182; BD Biosciences, San Jose, CA, USA). Secondary antibodies (Jackson ImmunoResearch Laboratories, West Grove, PA, USA) used were donkey anti-rabbit Cyanine Cy3 AffiniPure IgG (Cat. No. 711-165-152) for ZO-1, Alexa Fluor-488 AffiniPure F(ab’)_2_ Fragment IgM (Cat. No. 715-546-020) for vimentin, and donkey anti-rabbit Alexa Fluor-647 AffiniPure F(ab’)_2_ fragment (Cat. No. 715-606-150) for E-cadherin. Other reagents used for immunocytochemistry analysis included paraformaldehyde, Tween 20, bovine serum albumin, glutaraldehyde, Triton X-100, sodium chloride, and DAPI (all from Sigma-Aldrich), normal donkey serum (Cat. No. 017-000-121; Jackson ImmunoResearch Laboratories), and Fluoro-Gel mounting medium (Electron Microscopy Sciences; Hatfield, PA, USA).

### 5.2. Scaffold Fabrication and Modification

Conventional electrospinning and cryoelectrospinning were performed using 1% *w*/*v* elastin, 1.5% *w*/*v* alginate, and 3% *w*/*v* PEG-400kD in deionized water, as detailed in our previous publication [19]. Briefly, 3% PEG-400kD was essential to facilitate chain-chain interaction of the polymer molecules, whereas 1% elastin and 1.5% alginate enabled the right viscosity for successful electrospinning of nanofibers. The biomaterial solution was electrospun at optimized parameters of 17 kV (needle voltage), 10 µL/min (flow rate), through a 25 G needle with a 15 cm tip-to-collector distance, to maintain a stable Taylor cone for electrospinning of nanofibers instead of electrospraying of droplets.

NF mats were electrospun under these parameters on a flat collector plate for 1 h at room temperature and <35% relative humidity (RH) to ensure homogeneous, bead-free nanofibers. CES-H and CES-F were cryoelectrospun using the same parameters but at >35% RH onto a custom probe-array collector maintained at −15 to −20 °C for 1 h to enable ice crystal formation between the deposited fibers and therefore 3D scaffold formation, as previously described [19]. The air temperature was modulated to >2 °C to fabricate CES-F or <2 °C to fabricate CES-H through solute–solvent phase separation at near-zero air temperatures. Temperature and humidity were continuously monitored to ensure reproducible fabrication of different scaffold types. After cryoelectrospinning, the collector with the scaffolds was immediately lyophilized in a FreeZone Freeze Dryer (Labconco, Kansas City, MI, USA) for 2–3 h.

Electrospun NF mats and lyophilized CES-F and CES-H were individually crosslinked in a 96-well plate using EDC and NHS (1.48 mg EDC + 1.78 mg NHS per 100 µL 95% ethanol per scaffold) to covalently link elastin and alginate chains. PEG-400kD does not have pendant groups that can be crosslinked and dissolves away in water in the subsequent wash steps. Scaffolds were rocked at 45 rpm for 2 h, then washed sequentially in 95%, 70%, 50%, and 0% *v*/*v* ethanol in deionized water containing 1.5% *w*/*v* CaCl_2_ for 15 min each to remove residual EDC and NHS and ionically crosslink alginate chains. Finally, scaffolds were frozen at −80 °C and lyophilized for 4 h.

All scaffolds were UV-sterilized, soaked in 70% *v*/*v* ethanol + 50 mM CaCl_2_ for 30 min, rinsed with 0.9% *w*/*v* NaCl + 50 mM CaCl_2_ for 10 min, and hydrated overnight in culture medium (10% FBS, 5% Antibiotic-Antimycotic Solution, 50 mM CaCl_2_) prior to cell seeding.

### 5.3. Scanning Electron Microscopy (SEM)

For SEM imaging of scaffolds without cells, electrospun NF mats and lyophilized cryoelectrospun CES-H and CES-F scaffolds were mounted on carbon taped metal stubs, prior to crosslinking to avoid hydration.

For SEM imaging of scaffolds seeded with cells, samples were fixed with 4% *w*/*v* paraformaldehyde–0.25% *v*/*v* glutaraldehyde in 5% (*w*/*v*) sucrose and 0.6× phosphate-buffered saline (PBS) for 20 min, followed by 3% *v*/*v* glutaraldehyde in 0.1 M sucrose–0.1 M phosphate buffer (pH 7.4) for 2 h. Fixed samples were washed three times (10 min each) in 0.1 M sucrose–0.1 M phosphate buffer, dehydrated through graded ethanol (25%, 50%, 70%, 80%, 95%, 100%, and 100% *v*/*v*) for 15 min per step, and chemically dried in ethanol/HMDS mixtures (3:1, 1:1, and 1:3 ethanol: HMDS) for 15 min each, followed by three rinses in 100% HMDS (15 min each). Samples were air-dried overnight and mounted on carbon taped metal stubs.

Scaffold samples with or without cells were sputter-coated with iridium-palladium and imaged using a Zeiss Leo 1550 field emission SEM (Zeiss Leo Electron Microscopy Ltd., Cambridge, UK).

### 5.4. Image Analysis of Scaffold Topographical Features

SEM images of NF, CES-H and CES-F scaffolds were analyzed in ImageJ (v1.54p) to quantify fiber diameter or backbone width and pore size. Measurements were taken from 50 to 100 features per image using the ‘Analyze > Measure’ tool.

### 5.5. Cell Culture

Mouse embryonic NIH 3T3 fibroblasts [33] (passage 12–17) and SIMS mouse submandibular salivary gland ductal epithelial cells [32] were cultured in DMEM containing 10% FBS and 1% penicillin–streptomycin at 37 °C, in a 5% CO_2_ humidified incubator. NIH 3T3 fibroblasts were subcultured on day 3 or 4 at 70–80% confluence and SIMS cells every 2 or 3 days at 80–95% confluence.

### 5.6. Cell Culture on Scaffolds

For epithelial monoculture, SIMS cells were seeded at 75,000 cells/scaffold in 25 μL DMEM (10% FBS, 1% penicillin-streptomycin, and 25 mM CaCl_2_) in ultra-low adhesion, Lipidure polymer-coated, round-bottom, 96-well plates. 25 mM CaCl_2_ supplementation was used to prevent rapid disintegration of the scaffold, a concentration at which cell health was not negatively impacted [8,19,21]. Immediately after cell seeding, the samples were transferred to a rotary shaker in an incubator and gently rotated at 30 rpm for 2 h to promote cell attachment to the scaffolds. After 2 h, 175 μL of fresh medium was added to each well and rotary shaking continued for an additional 22 h to increase cell attachment efficiency. After 24 h, the cell-scaffold constructs were switched to static culture by transfer to Lipidure-coated 24-well plates containing 200 μL fresh medium per well to improve the surface area/volume ratio, and thereby enhance oxygen transfer and cell viability [19]. Cells were maintained in static culture for 7 days with daily partial medium changes (removing 100 μL spent medium and adding 150 μL fresh medium) to partially retain conditioned medium while replenishing nutrients and compensating for evaporative losses.

For stromal-epithelial cocultures, NIH 3T3 cells and SIMS cells were seeded using the same procedure as described above by first seeding 50,000 NIH 3T3 cells, culturing for 2 days, followed by seeding 50,000 SIMS cells and continuing the culture for 4–7 days.

### 5.7. Immunochemistry Analysis and Confocal Imaging of Cell-Scaffold Constructs

Cell-scaffold constructs were fixed in 4% *w*/*v* paraformaldehyde–0.25% *v*/*v* glutaraldehyde in 5% (*w*/*v*) sucrose, 0.6× PBS for 15 min, permeabilized with 0.1% *v*/*v* Triton X-100 in 1× PBS for 15 min, and blocked with 20% *v*/*v* donkey serum–3% *w*/*v* bovine serum albumin in wash buffer (0.9% *w*/*v* NaCl–50 mM CaCl_2_ in deionized water) for 2 h at room temperature. Samples were incubated overnight at 4 °C with primary antibodies, mouse µ-chain specific anti-vimentin (clone LN-6, 1:400 dilution), rabbit polyclonal anti-ZO-1 (1:300 dilution) and mouse monoclonal anti-E-cadherin (1:300 dilution), followed by 2 h, room-temperature incubation with DAPI (1:400 dilution) and secondary antibodies, donkey anti-rabbit Cyanine Cy3 (to reveal ZO-1, 1:400 dilution), donkey anti-mouse IgM Alexa Fluor-488 (to reveal vimentin, 1:400 dilution), and donkey anti-mouse IgG Alexa Fluor-647 (to reveal E-cadherin, 1:400 dilution). NIH 3T3 fibroblasts were immunostained for vimentin and SIMS epithelial cells were stained for E-cadherin and ZO-1. All samples were counterstained with DAPI and mounted in Fluoro-Gel mounting medium for confocal imaging using a Leica SP5 confocal laser scanning microscope (Leica Microsystems, Mannheim, Germany).

## 6. Patents

Pujhitha Ramesh, James Castracane, Melinda Larsen, Susan Sharfstein, and Yubing Xie have pending patent applications (US Patent App. 17/558,543 and 18/108,395) related to this work.

## Figures and Tables

**Table 1:** Summary of scaffold topographical features.

Scaffold Type	Minimum Fiber Diameter or Backbone Width	Maximum Fiber Diameter or Backbone Width	Average Fiber Diameter or Backbone Width	Pore Size
NF	108 nm	329 nm	200 nm ± 54 nm	<2 µm
CES-F	62 nm	363 nm	178 nm ± 80 nm	<5 µm
CES-H	980 nm	28 µm	4.72 ± 3.86 µm	15–25 µm

## Data Availability

The original contributions presented in this study are included in this article; further inquiries can be directed to the corresponding authors.

## Abbreviations

The following abbreviations are used in this manuscript:

CES	Elastin-alginate cryoelectrospun scaffolds
CES-F	Elastin-alginate cryoelectrospun scaffolds with fibrous topography
CES-H	Elastin-alginate cryoelectrospun scaffolds with honeycomb topography
DAPI	Diamidino-2-phenylindole
DMEM	Dulbecco’s modified eagle medium
ECM	Extracellular matrix
FBS	Fetal bovine serum
HMDS	Hexamethyldisilazane
MSC	Mesenchymal stromal cell
NFs	Conventionally electrospun nanofibers
PBS	Phosphate buffered saline
PEG	Poly(ethylene glycol)
SEM	Scanning electron microscopy
ZO-1	Zona occludin-1

## References

[1] Krishnamurthy S., Vasudeva S.B., Vijayasarathy S. (2015). Salivary Gland Disorders: A Comprehensive Review. World J. Stomatol..

[2] Vistoso Monreal A., Polonsky G., Shiboski C., Sankar V., Villa A. (2022). Salivary Gland Dysfunction Secondary to Cancer Treatment. Front. Oral Health.

[3] Tanaka J., Mishima K. (2020). In Vitro Three-Dimensional Culture Systems of Salivary Glands. Pathol. Int..

[4] Burghartz M., Lennartz S., Schweinlin M., Hagen R., Kleinsasser N., Hackenberg S., Steußloff G., Scherzad A., Radeloff K., Ginzkey C. (2018). Development of Human Salivary Gland-Like Tissue In Vitro. Tissue Eng. Part A.

[5] Kim D., Yoon Y.J., Choi D., Kim J., Lim J.Y. (2021). 3D Organoid Culture From Adult Salivary Gland Tissues as an Ex Vivo Modeling of Salivary Gland Morphogenesis. Front. Cell Dev. Biol..

[6] Sui Y., Zhang S., Li Y., Zhang X., Hu W., Feng Y., Xiong J., Zhang Y., Wei S. (2020). Generation of Functional Salivary Gland Tissue from Human Submandibular Gland Stem/Progenitor Cells. Stem Cell Res. Ther..

[7] Ozdemir T., Fowler E.W., Hao Y., Ravikrishnan A., Harrington D.A., Witt R.L., Farach-Carson M.C., Pradhan-Bhatt S., Jia X. (2016). Biomaterials-Based Strategies for Salivary Gland Tissue Regeneration. Biomater. Sci..

[8] Jorgensen M., Ramesh P., Toro M., Evans E., Moskwa N., Zhang X., Sharfstein S.T., Larsen M., Xie Y. (2022). Alginate Hydrogel Microtubes for Salivary Gland Cell Organization and Cavitation. Bioengineering.

[9] Shin H.S., Hong H.J., Koh W.G., Lim J.Y. (2018). Organotypic 3D Culture in Nanoscaffold Microwells Supports Salivary Gland Stem-Cell-Based Organization. ACS Biomater. Sci. Eng..

[10] Gluck C., Min S., Oyelakin A., Smalley K., Sinha S., Romano R.A. (2016). RNA-Seq Based Transcriptomic Map Reveals New Insights into Mouse Salivary Gland Development and Maturation. BMC Genom..

[11] Su X., Fang D., Liu Y., Ramamoorthi M., Zeitouni A., Chen W., Tran S.D. (2016). Three-Dimensional Organotypic Culture of Human Salivary Glands: The Slice Culture Model. Oral Dis..

[12] Phan T.V., Oo Y., Ahmed K., Rodboon T., Rosa V., Yodmuang S., Ferreira J.N. (2023). Salivary Gland Regeneration: From Salivary Gland Stem Cells to Three-Dimensional Bioprinting. SLAS Technol..

[13] Maruyama C.L., Monroe M.M., Hunt J.P., Buchmann L., Baker O.J. (2018). Comparing Human and Mouse Salivary Glands: A Practice Guide for Salivary Researchers. Oral Dis..

[14] Frangogiannis N.G. (2022). Why Animal Model Studies Are Lost in Translation. J. Cardiovasc. Aging.

[15] Aisenbrey E.A., Murphy W.L. (2020). Synthetic Alternatives to Matrigel. Nat. Rev. Mater..

[16] Revilla S.A., Cutilli A., Cambiaso E., Rockx-Brouwer D., Frederiks C.L., Falandt M., Levato R., Kranenburg O., Lindemans C.A., Coffer P.J. (2025). Impact of 3D Cell Culture Hydrogels Derived from Basement Membrane Extracts or Nanofibrillar Cellulose on CAR-T Cell Activation. iScience.

[17] Valdoz J.C., Johnson B.C., Jacobs D.J., Franks N.A., Dodson E.L., Sanders C., Cribbs C.G., Van Ry P.M. (2021). The ECM: To Scaffold, or Not to Scaffold, That Is the Question. Int. J. Mol. Sci..

[18] Andrews M.G., Kriegstein A.R. (2022). Challenges of Organoid Research. Annu. Rev. Neurosci..

[19] Ramesh P., Moskwa N., Hanchon Z., Koplas A., Nelson D.A., Mills K.L., Castracane J., Larsen M., Sharfstein S.T., Xie Y. (2022). Engineering Cryoelectrospun Elastin-Alginate Scaffolds to Serve as Stromal Extracellular Matrices. Biofabrication.

[20] Ramesh P. (2022). Biomimetic Scaffolds Targeting Remediation of Fibrosis and Regeneration of the Salivary Gland.

[21] Ramesh P., Pena R., Morrissey J.M., Moskwa N., Tubbesing K., Zhang X., Nelson D., Castracane J., Khmaladze A., Sharfstein S.T. (2025). Cryoelectrospun Elastin-Alginate Scaffolds as Potential Cell Delivery Vehicles for Mesenchymal Stromal Cell Therapy. Sci. Rep..

[22] Moskwa N., Mahmood A., Nelson D.A., Altrieth A.L., Forni P.E., Larsen M. (2022). Single-Cell RNA Sequencing Reveals PDFGRα+ Stromal Cell Subpopulations That Promote Proacinar Cell Differentiation in Embryonic Salivary Gland Organoids. Development.

[23] Hosseini Z.F., Nelson D.A., Moskwa N., Sfakis L.M., Castracane J., Larsen M. (2018). FGF2-Dependent Mesenchyme and Laminin-111 Are Niche Factors in Salivary Gland Organoids. J. Cell Sci..

[24] Salimbeigi G., Vrana N.E., Ghaemmaghami A.M., Huri P.Y., McGuinness G.B. (2022). Basement Membrane Properties and Their Recapitulation in Organ-on-Chip Applications. Mater. Today Bio.

[25] Kozyrina A.N., Piskova T., Di Russo J. (2020). Mechanobiology of Epithelia from the Perspective of Extracellular Matrix Heterogeneity. Front. Bioeng. Biotechnol..

[26] Frantz C., Stewart K.M., Weaver V.M. (2010). The Extracellular Matrix at a Glance. J. Cell Sci..

[27] Bosman F.T., Stamenkovic I. (2003). Functional Structure and Composition of the Extracellular Matrix. J. Pathol..

[28] Lipp S.N., Jacobson K.R., Hains D.S., Schwarderer A.L., Calve S. (2021). 3D Mapping Reveals a Complex and Transient Interstitial Matrix During Murine Kidney Development. J. Am. Soc. Nephrol..

[29] Beucler M.J., Miller W.E. (2019). Isolation of Salivary Epithelial Cells from Human Salivary Glands for In Vitro Growth as Salispheres or Monolayers. J. Vis. Exp..

[30] Jeong Y.J., Hong Y., Yoon Y.J., Sim N.S., Hong S.M., Lim J.Y. (2025). Chemical Reprogramming Culture for the Expansion of Salivary Gland Epithelial Basal Progenitor Cells. Stem Cell Res. Ther..

[31] Athwal H.K., Lombaert I.M.A. (2019). 3D Organoid Formation from the Murine Salivary Gland Cell Line SIMS. Bio Protoc..

[32] Laoide B.M., Courty Y., Gastinne I., Thibaut C., Kellermann O., Rougeon F. (1996). Immortalised Mouse Submandibular Epithelial Cell Lines Retain Polarised Structural and Functional Properties. J. Cell Sci..

[33] Jainchill J.L., Aaronson S.A., Todaro G.J. (1969). Murine Sarcoma and Leukemia Viruses: Assay Using Clonal Lines of Contact-Inhibited Mouse Cells. J. Virol..

[34] Sharfstein S., Xie Y., Ramesh P., Castracane J., Larsen M., Mosk N. (2024). Compositions, Apparatuses and Methods for Making and Using Bioscaffolds.

[35] de Paula F., Teshima T.H.N., Hsieh R., Souza M.M., Nico M.M.S., Lourenco S.V. (2017). Overview of Human Salivary Glands: Highlights of Morphology and Developing Processes. Anat. Rec..

[36] Campbell H.K., Maiers J.L., DeMali K.A. (2017). Interplay Between Tight Junctions & Adherens Junctions. Exp. Cell Res..

[37] Danielsson F., Peterson M., Caldeira Araújo H., Lautenschläger F., Gad A. (2018). Vimentin Diversity in Health and Disease. Cells.

[38] Trappmann B., Gautrot J.E., Connelly J.T., Strange D.G.T., Li Y., Oyen M.L., Cohen Stuart M.A., Boehm H., Li B., Vogel V. (2012). Extracellular-Matrix Tethering Regulates Stem-Cell Fate. Nat. Mater..

[39] Sonbol H. (2018). Extracellular Matrix Remodeling in Human Disease. J. Microsc. Ultrastruct..

[40] Bonnans C., Chou J., Werb Z. (2014). Remodelling the Extracellular Matrix in Development and Disease. Nat. Rev. Mol. Cell Biol..

[41] Kwon S., Ryu J.H., Kim J., Shin H.H., Chung G., Taghizadeh A., Lee J.-H., Kim J., Ku B.-C., Park K. (2025). Biomimetic Catechol-Incorporated Polyacrylonitrile Nanofiber Scaffolds for Tissue Engineering of Functional Salivary Glands. Biomater. Res..

[42] Song Y., Uchida H., Sharipol A., Piraino L., Mereness J.A., Ingalls M.H., Rebhahn J., Newlands S.D., DeLouise L.A., Ovitt C.E. (2021). Development of a Functional Salivary Gland Tissue Chip with Potential for High-Content Drug Screening. Commun. Biol..

[43] Pradhan-Bhatt S., Harrington D.A., Duncan R.L., Jia X., Witt R.L., Farach-Carson M.C. (2013). Implantable Three-Dimensional Salivary Spheroid Assemblies Demonstrate Fluid and Protein Secretory Responses to Neurotransmitters. Tissue Eng. Part A.

[44] Foraida Z.I., Kamaldinov T., Nelson D.A., Larsen M., Castracane J. (2017). Elastin-PLGA Hybrid Electrospun Nanofiber Scaffolds for Salivary Epithelial Cell Self-Organization and Polarization. Acta Biomater..

[45] Wells K.L., Gaete M., Matalova E., Deutsch D., Rice D., Tucker A.S. (2013). Dynamic Relationship of the Epithelium and Mesenchyme during Salivary Gland Initiation: The Role of Fgf10. Biol. Open.

[46] Sathi G.A., Farahat M., Hara E.S., Taketa H., Nagatsuka H., Kuboki T., Matsumoto T. (2017). MCSF Orchestrates Branching Morphogenesis in Developing Submandibular Gland Tissue. J. Cell Sci..

[47] Marinkovic M., Tran O.N., Wang H., Abdul-Azees P., Dean D.D., Chen X.D., Yeh C.K. (2023). Extracellular Matrix Turnover in Salivary Gland Disorders and Regenerative Therapies: Obstacles and Opportunities. J. Oral Biol. Craniofac. Res..

[48] Bissell D.M. (2001). Chronic Liver Injury, TGF-β, and Cancer. Exp. Mol. Med..

[49] Hupfer A., Brichkina A., Koeniger A., Keber C., Denkert C., Pfefferle P., Helmprobst F., Pagenstecher A., Visekruna A., Lauth M. (2021). Matrix Stiffness Drives Stromal Autophagy and Promotes Formation of a Protumorigenic Niche. Proc. Natl. Acad. Sci. USA.

[50] O’Connor J.W., Gomez E.W. (2014). Biomechanics of TGFβ-Induced Epithelial-Mesenchymal Transition: Implications for Fibrosis and Cancer. Clin. Transl. Med..

[51] Liu P., Zhang D., Huang G., Xue M., Fang Y., Lu L., Zhang J., Xie M., Ye Z. (2023). Laminin A1 as a Target for the Treatment of Epidural Fibrosis by Regulating Fibrotic Mechanisms. Int. J. Mol. Med..

[52] Peters S.B., Naim N., Nelson D.A., Mosier A.P., Cady N.C., Larsen M. (2014). Biocompatible Tissue Scaffold Compliance Promotes Salivary Gland Morphogenesis and Differentiation. Tissue Eng. Part A.

